# Fused Ischiorectal Phlegmon with Pre- and Retroperitoneal Extension: Case Report and Narrative Literature Review

**DOI:** 10.3390/jcm14144959

**Published:** 2025-07-13

**Authors:** Laurențiu Augustus Barbu, Liviu Vasile, Liliana Cercelaru, Ionică-Daniel Vîlcea, Valeriu Șurlin, Stelian-Stefaniță Mogoantă, Gabriel Florin Răzvan Mogoș, Tiberiu Stefăniță Țenea Cojan, Nicolae-Dragoș Mărgăritescu

**Affiliations:** 1 Department of Surgery, Railway Clinical Hospital Craiova, University of Medicine and Pharmacy of Craiova, 2 Petru Rares Street, 200349 Craiova, Romania; laurentiu.barbu@umfcv.ro (L.A.B.); gabriel.mogos@umfcv.ro (G.F.R.M.); 2 Department of Surgery, Emergency County Hospital, University of Medicine and Pharmacy of Craiova, 2 Petru Rares Street, 200349 Craiova, Romania; vliviu777@yahoo.com (L.V.); ionica.vilcea@umfcv.ro (I.-D.V.); vsurlin@gmail.com (V.Ș.); ssmogo@yahoo.com (S.-S.M.); dmargaritescu@yahoo.com (N.-D.M.); 3 Department of Embryology and Anatomy, University of Medicine and Pharmacy of Craiova, 200349 Craiova, Romania; liliana.cercelaru@umfcv.ro

**Keywords:** horseshoe ischiorectal abscess, extraperitoneal extension, retroperitoneal spread, perirectal sepsis, emergency laparotomy, necrotizing infection

## Abstract

**Background/Objectives:** Anorectal and retroperitoneal abscesses, although differing in frequency and presentation, present significant diagnostic and therapeutic challenges, especially when interconnected through complex fascial planes. Rare cases such as horseshoe ischiorectal phlegmons with extraperitoneal spread are particularly difficult to manage due to limited literature and the absence of standardized protocols. This article presents a rare case alongside a narrative review of similar cases, aiming to highlight key diagnostic pitfalls and therapeutic strategies. **Methods:** We conducted a narrative literature review using PubMed, Embase, Scopus, and Web of Science to identify reports on horseshoe ischiorectal phlegmons with extraperitoneal or retroperitoneal extension. Relevant studies were compared with the present case. **Results:** We describe a 59-year-old male who presented with severe sepsis, diffuse abdominal pain, and hemodynamic instability. Imaging and surgery revealed extensive necrotizing spread to the anterior abdominal wall, peritoneum, and retroperitoneal space, despite absent local perianal signs. Emergency midline laparotomy, wide debridement, and drainage were performed. Despite intensive care, the patient suffered rapid clinical deterioration and died within six hours postoperatively. **Conclusions:** This case and literature review highlight how a clinically silent ischiorectal phlegmon can progress to extensive extraperitoneal involvement and fatal sepsis. This underscores the need for early recognition, advanced imaging, and aggressive multidisciplinary management. Further studies are needed to develop evidence-based guidelines for complex anorectal abscesses with deep fascial extension.

## 1. Introduction

Anorectal abscesses are among the most common proctologic emergencies, with an estimated 70,000 to 90,000 cases reported annually in the United States. They typically result from obstruction or infection of the anal glands, often associated with risk factors such as chronic constipation, inflammatory bowel disease, malignancy, foreign bodies, or sexually transmitted infections [1]. According to Parks et al. [2], anorectal abscesses are classified into perianal, ischiorectal, intersphincteric, and supralevator types, with supralevator abscesses being the rarest, accounting for only 1–9% of cases. These deep abscesses often present with non-specific symptoms, including anal pain and fever, frequently without visible perineal signs [2].

Retroperitoneal abscesses are rare but serious infections that often follow a silent, insidious course, contributing to delayed diagnosis and increased risks of sepsis and mortality, which may reach 11–20% in reported series [3,4,5,6]. Horseshoe abscesses, a complex variant arising from perirectal infections, can extend bilaterally across the ischiorectal fossae and, in rare cases, spread into retroperitoneal spaces. Such extensive disease poses significant diagnostic and surgical challenges and is associated with a high risk of recurrence and complications.

Understanding retroperitoneal fascial anatomy is crucial for explaining the pathways of disease spread. While classical descriptions such as the Zuckerkandl and Gerota fasciae have long been recognized, modern imaging and anatomical studies have revealed that the retroperitoneal fascial planes are more complex than the traditional tricompartmental (TC) model suggests. The newer concept of the combined interfascial spread (CIS) model emphasizes the existence of potential spaces and interfascial planes that may facilitate the dissemination of infection [7].

Perirectal abscesses rarely extend beyond the pelvic compartment; however, when they spread to extraperitoneal spaces—such as the prevesical, presacral, or Retzius space—diagnosis is often delayed due to vague, nonspecific symptoms and the deep anatomical location [8]. These unusual patterns of spread demand a high index of suspicion, advanced imaging, and individualized management strategies.

Given the rarity of cases with extensive extraperitoneal and retroperitoneal extension and the lack of clear management guidelines, we present a rare and severe case of a horseshoe ischiorectal phlegmon with both pre- and retroperitoneal spread, accompanied by a narrative literature review. This combined approach aims to illustrate key anatomical and clinical aspects, compare similar cases, and highlight diagnostic pitfalls and surgical considerations that may improve outcomes in such complex presentations.

## 2. Case Presentation

### 2.1. Patient Information

We report the case of a 59-year-old male with no known significant comorbidities such as diabetes mellitus or chronic liver disease. He had a history of smoking (approximately 20 pack-years) and lived in a rural area with limited access to healthcare services. There was no relevant personal or family history of colorectal or perianal disease. Written informed consent for emergency treatment was obtained from the patient, and the patient’s family provided written consent for the use of anonymized data in this report.

### 2.2. Clinical Findings

The patient presented to the emergency department with diffuse abdominal pain lasting for three days, accompanied by high-grade fever, chills, and marked deterioration of his general condition.

On physical examination, the abdomen was moderately distended with diffuse tenderness and generalized guarding, consistent with signs of peritoneal irritation. Digital rectal examination was unremarkable, with normal findings of the ischiorectal fossae and overlying skin.

Initial vital signs revealed hypotension (blood pressure: 95/60 mmHg), tachycardia (heart rate: 118 beats per minute), tachypnea (respiratory rate: 24 breaths per minute), hyperthermia (temperature: 39.2 °C), and an oxygen saturation of 92% on room air.

### 2.3. Timeline

The clinical evolution of the patient is summarized in Table 1, detailing the key events from symptom onset to the final outcome. The timeline highlights the rapid progression of the condition and the critical interventions undertaken.

### 2.4. Diagnostic Assessment

**Figure 1:**
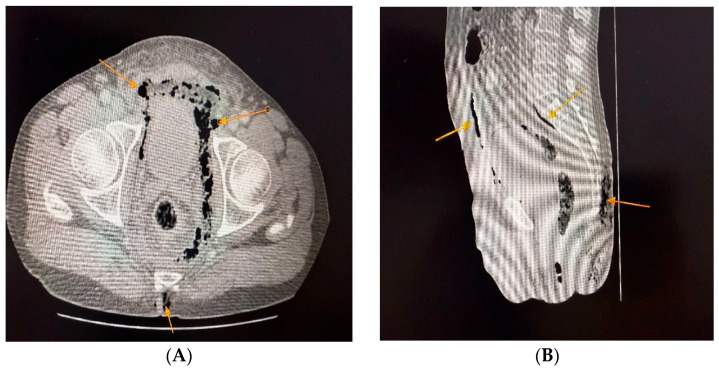
The CT scan demonstrates evidence of pro- and retropneumoperitoneum (**A-yellow arrow**), as well as a loculated collection with gas within the ischiorectal fossa, suggestive of an advanced infectious process with tissue necrosis (**B-yellow arrow**).

**Figure 2:**
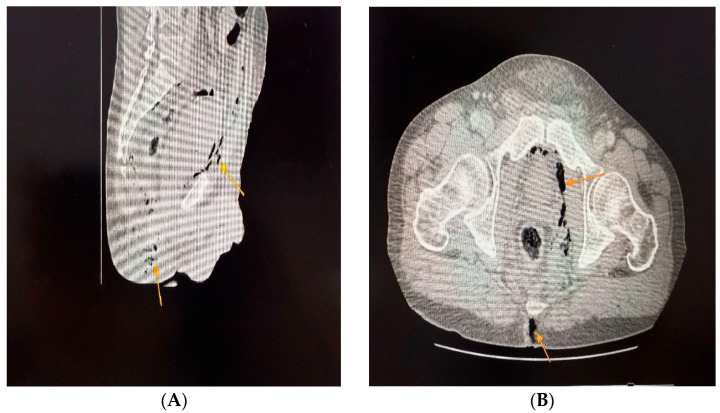
Computed tomography demonstrated anterior pneumoperitoneum (**A-yellow arrow**) and identified a gas-containing collection within the ischiorectal fossa, findings suggestive of an ongoing deep-seated infectious process (**B-yellow arrow**).

**Figure 3:**
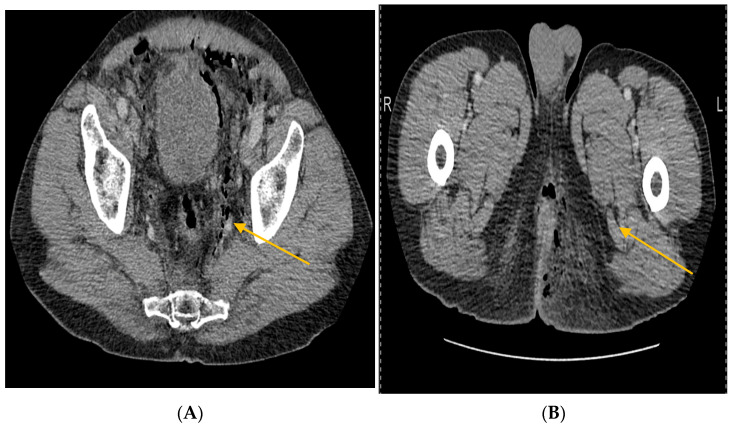
Axial CT images show bilateral ischiorectal phlegmonous collections with preperitoneal and retroperitoneal extension and free air (**A-yellow arrow**), and perineal soft tissue swelling with air pockets in both ischiorectal fossae, suggesting anaerobic infection with extensive necrosis (**B-yellow arrow**).

- Labs: marked leukocytosis (19,200/µL), neutrophilia (89.8%), thrombocytopenia, renal dysfunction (creatinine 1.7 mg/dL, urea 93 mg/dL), elevated fibrinogen, C-reactive protein, ESR, procalcitonin, and serum lactate (Table 2).
- Imaging: CT showed bilateral ischiorectal phlegmon with air in preperitoneal and retroperitoneal spaces → anterior/posterior pneumoperitoneum (Figure 1, Figure 2 and Figure 3). CT imaging was performed at the Radiology Department, Emergency County Hospital Slatina, using a SOMATOM Definition AS scanner (Siemens Healthineers, Erlangen, Germany).
- Microbiology: intraoperative purulent fluid collected for cultures and antibiogram (results pending at time of death).
- Differential diagnosis: deep perianal sepsis (Fournier’s gangrene), intra-abdominal perforation, necrotizing fasciitis.
- Definitive diagnosis: advanced necrotizing ischiorectal sepsis with retroperitoneal extension.

### 2.5. Therapeutic Intervention

**Figure 4:**
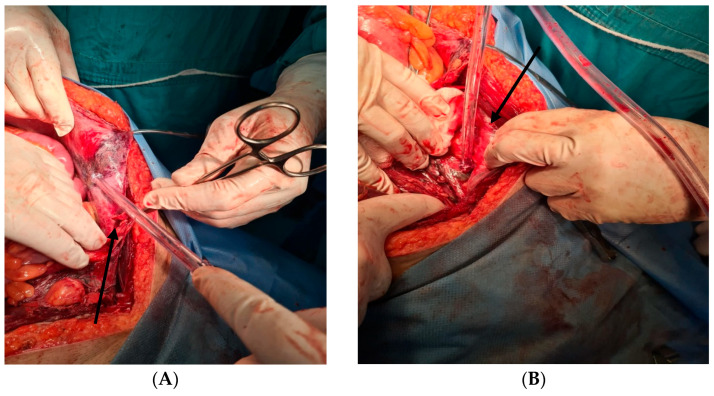
The intraoperative findings revealed extensive areas of peritoneal necrosis (**A-black arrow**), accompanied by muscular necrosis of the rectus abdominis muscles (**B-black arrow**), indicative of a severe and advanced infectious process with transmural involvement of the abdominal wall structures.

- Emergency midline laparotomy: drainage of purulent fluid, debridement of necrotic rectus abdominis and anterior parietal peritoneum, extensive retroperitoneal debridement, multiple drains placed (Figure 4).
- Medical management: broad-spectrum IV antibiotics (imipenem 500 mg q6 h + vancomycin 1 g q12 h), IV fluids, analgesia (morphine 2 mg/h), antipyretics, electrolyte correction, organ support.
- Rationale: empirical broad-spectrum coverage due to severe sepsis with deep tissue involvement.

### 2.6. Follow-Up and Outcomes

Despite aggressive surgical and medical management, the patient’s condition worsened rapidly. He developed multi-organ failure and suffered a cardiorespiratory arrest 6 h postoperatively, unresponsive to advanced resuscitation measures.

### 2.7. Patient Perspective

Due to the fatal outcome, a direct patient perspective is unavailable. The patient’s family was informed about the severity of the condition and the poor prognosis and consented to the use of anonymized data for publication.

### 2.8. Informed Consent

Written informed consent for publication was obtained from the patient’s next of kin.

## 3. Discussion

Deep perianal abscesses represent rare but serious anorectal conditions that carry a significant risk of delayed diagnosis, progression to sepsis, and increased mortality, particularly in patients with comorbidities such as inflammatory bowel disease (IBD), cardiovascular disease, or diabetes mellitus. Their atypical presentation can mimic intra-abdominal conditions, further complicating timely clinical recognition and intervention.

Retroperitoneal abscesses most commonly originate from genitourinary infections and are associated with poor outcomes when diagnosis and treatment are delayed, with reported mortality rates reaching up to 26%. The supralevator space, situated above the levator ani muscle, communicates with multiple pelvic and abdominal compartments, providing potential pathways for anterior and posterior spread when normal anatomical barriers are disrupted, often through the formation of fistulous tracts. A detailed understanding of pelvic and retroperitoneal anatomy is therefore essential to improve diagnostic accuracy and guide appropriate management in such complex and rare cases [6].

According to the cryptoglandular theory, anorectal abscesses originate from obstruction and infection of the anal glands located near the dentate line. The infection initially spreads intersphincterically and can extend into the perianal or ischiorectal spaces [9,10,11]. In rare cases, the infection may ascend, leading to supralevator abscess formation. Although perianal and ischiorectal abscesses represent the most common forms, deeper variants—such as deep postanal, horseshoe, and supralevator abscesses—can occur, necessitating precise anatomical knowledge for appropriate surgical management. Differential diagnoses include inflammatory bowel disease, trauma, malignancies, and uncommon infections such as tuberculosis [11].

The ischiorectal fossa, bordered by the pubic ramus, gluteus maximus, and obturator fascia, contains loose connective tissue that is highly susceptible to infection and provides communication pathways to adjacent pelvic and subperitoneal spaces. This anatomical continuity facilitates the potential spread of infection and increases the risk of peritonitis [7].

The retroperitoneal fasciae develop through three main embryological mechanisms: the extension of connective tissue sheaths from the abdominal wall musculature, the fusion of peritoneal layers during retroperitoneal organ fixation (e.g., Toldt’s fascia), and the alignment of connective fibers due to organ growth tension, as seen in the renal fascia [12,13,14,15]. Based on these anatomical structures, Meyers’ tricompartmental concept (1972) subdivided the retroperitoneum into perirenal, anterior pararenal, and posterior pararenal compartments, along with a fourth compartment encompassing the “great vessels” such as the aorta and inferior vena cava [12,13,16]. While the lateroconal fascia was traditionally believed to separate these spaces, recent imaging and embryological studies suggest that fluid and infection can cross these planes freely, supporting the concept of a continuous interfascial interface defined more by lateral fat pads than by distinct fascia fusion [7,17,18].

The concept of Interfascial Spread (CIS), as proposed by Molmenti et al. [19], reframes our understanding of retroperitoneal disease dissemination by defining potential pathways—namely the retromesenteric, retrorenal, lateroconal, and conjoined interfascial planes—located between fascial layers rather than within fixed anatomical compartments. This model addresses the limitations of the traditional tricompartmental concept, particularly in explaining posterior and pelvic extensions [19]. However, the CIS framework relies predominantly on radiological evidence, and its direct anatomical validation remains limited [19].

While the CIS hypothesis suggests that these planes result from the embryological fusion of secondary retroperitoneal organs with the posterior abdominal wall [19,20,21,22], Ishikawa et al. have argued that they may instead represent residual primitive mesenchymal connective tissue [23]. This alternative perspective redefines the retromesenteric, lateroconal, and retrorenal regions as loosely organized connective tissue zones between fascial and peritoneal layers, which is more consistent with observed disease spread patterns [18]. Furthermore, the idea of a continuous “combined interfascial plane” has been questioned, since the renal fascia caudal to the kidney is continuous and envelops structures such as the ureters, gonadal vessels, and perinephric veins, directing fluid to spread laterally rather than through a single unified pathway [18,24,25].

Most perirenal fat originates from connective tissue embryologically associated with the gonads and adrenal glands, whereas only a small portion adjacent to the renal hilum and periureteral region is linked to the urinary system and is enclosed within a distinct “ureteral sheath.” This detailed compartmentalization challenges the notion of continuous interfascial planes, as secondary retroperitoneal organs develop at different embryonic stages and are delineated by specific fusion fasciae, including Toldt’s, Treitz’s, and Fredet’s fasciae [22,24,26,27]. These fusion planes act as potential anatomical pathways for disease spread between residual parietal and visceral peritoneum, especially behind the pancreas, duodenum, and colon, extending anteriorly via the fascia of Fredet [7]. The retropancreatic fascia, which forms during fetal development through mesenteric fusion and tissue migration, serves both as a conduit for disease extension and as a barrier—particularly against pancreatic tumor invasion—by outlining the peripancreatic space, which can direct inflammatory or neoplastic processes into adjacent mesenteric and retroperitoneal regions, as observed in pancreatitis [15,28,29,30]. However, the variable anatomy of this fascia, especially near the superior mesenteric vessels, facilitates perivascular and neural invasion, highlighting the need for detailed radiological assessment to accurately map retroperitoneal disease patterns [31].

The concept of Interfascial Spread (CIS), introduced by Molmenti et al., offers a refined perspective on retroperitoneal disease dissemination by defining potential planes—retromesenteric, retrorenal, lateroconal, and conjoined interfascial spaces—located between fascial layers rather than within rigid anatomical compartments [19]. This model addresses gaps in the traditional tricompartmental approach, particularly for explaining posterior and pelvic spread. However, CIS remains primarily based on radiological interpretations and lacks complete anatomical validation [19].

While CIS proposes that these planes arise through embryological fusion of secondary retroperitoneal organs with the posterior abdominal wall [19,20,21,22], Ishikawa et al. argue they represent residual primitive mesenchymal connective tissue instead [23]. This alternative view describes the retromesenteric, lateroconal, and retrorenal regions as loose connective tissue zones located between fascial and peritoneal layers, which aligns more closely with observed patterns of disease extension [18]. Additionally, the idea of a single continuous “combined interfascial plane” is debated, since the renal fascia below the kidney is continuous and envelops the ureters, gonadal vessels, and perinephric veins, directing fluid spread laterally instead of through a unified plane [18,24,25].

Most perirenal fat originates from connective tissue embryologically linked to the gonads and adrenal glands, while only a small portion near the renal hilum and periureteral region is associated with the urinary system and is enclosed within a distinct “ureteral sheath.” This detailed segmentation challenges the CIS assumption of continuous interfascial spread, as secondary retroperitoneal organs develop at different embryonic stages and are separated by defined fusion fasciae, including Toldt’s, Treitz’s, and Fredet’s fasciae [22,24,26,27]. These fusion planes create potential pathways for disease extension between parietal and visceral peritoneum, especially behind the pancreas, duodenum, and colon, extending anteriorly through Fredet’s fascia [7]. The retropancreatic fascia, which forms during fetal development through mesenteric fusion, acts both as a barrier and as a channel for disease spread—particularly relevant for pancreatic tumors—by delineating the peripancreatic space, which can guide inflammatory or neoplastic processes into surrounding mesenteric and retroperitoneal compartments, as seen in pancreatitis [15,28,29,30]. Its variable anatomy, especially near the superior mesenteric vessels, facilitates perivascular and neural invasion, emphasizing the importance of detailed imaging for accurately identifying retroperitoneal disease patterns [31].

Retroperitoneal necrotizing fasciitis (RNF) demands a high index of suspicion due to its nonspecific symptoms and rapid progression along fascial planes. Early diagnosis, guided by clinical signs—such as severe, disproportionate pain, signs of septic shock—combined with imaging and laboratory tests, is critical because infection can extend through connected retroperitoneal spaces when fascial barriers are compromised [9,32,33,34,35,36,37,38].

Several non-anorectal conditions can mimic anorectal abscesses without direct involvement of the anal canal. These include subcutaneous abscesses, carbuncles, infected sebaceous cysts, pilonidal abscesses in the midline gluteal cleft, and hidradenitis suppurativa, which may present with nodules, draining tracts, and scar tissue [39,40,41,42,43,44,45,46,47,48]. The differential diagnosis for perianal pain also includes thrombosed external hemorrhoids, acute anal fissures, perianal Crohn’s disease, and malignancy—highlighting the need for careful clinical evaluation, as up to 2.9% of new anorectal abscesses reveal underlying Crohn’s disease [11,49].

For unclear or complex abscesses, imaging is indicated because routine CT can miss early lesions; MRI and ultrasound offer better soft tissue detail and help define anatomy for targeted treatment [50,51,52]. CT remains the most sensitive method for diagnosing RNF by detecting fascial thickening and edema, though gas formation may be absent. While MRI provides excellent soft tissue contrast, it may risk overdiagnosis. Therefore, strong clinical suspicion remains essential, as imaging and scoring systems cannot replace timely surgical judgment.

Effective RNF management requires urgent, repeated surgical debridement and broad-spectrum antibiotics to fully eliminate necrotic tissue [47]. Horseshoe abscesses—which represent up to 20% of anorectal fistulas—spread circumferentially via Colles’ fascia and have high recurrence rates (18–50%) due to incomplete drainage or undetected fistulous tracts [46,53]. RNF should be suspected in patients with severe, rapidly spreading infections; early recognition based on clinical signs, imaging, and laboratory results is crucial due to the risk of widespread retroperitoneal extension [39,42,54].

Hyperbaric oxygen therapy (HBO) can support necrotizing fasciitis treatment by enhancing immune response and reducing bacterial toxin production; however, it may delay surgical intervention and limit monitoring, so repeated surgical debridement every 24–48 h and negative-pressure wound therapy remain standard practice. RNF carries a high mortality rate (10–50%), mainly due to delayed diagnosis and intervention [40,42,55], but early detection, aggressive surgery, and coordinated perioperative care can significantly improve outcomes [47].

Standard management of anorectal abscesses remains prompt surgical incision and drainage, preceded by assessment of sphincter function, relevant history, anticoagulation status, and comorbidities [56]. Small perianal or limited ischiorectal abscesses may be drained under local anesthesia in the emergency room, while larger or more complex cases require operating room management to ensure complete drainage and minimize recurrence [11]. Notably, previous reports have described horseshoe ischiorectal abscesses with extraperitoneal extension managed with open drainage combined with colostomy and fistulotomy, or, in delayed presentations, with drainage alone [45]. In our case, extensive midline laparotomy with additional preperitoneal and retroperitoneal incisions and multiple drains was necessary due to advanced disease spread.

The variation in therapeutic approaches reported across different cases highlights the lack of standardized clinical guidelines and emphasizes the need for evidence-based strategies that consider patient comorbidities and the complex anatomical features involved (Table 3).

The standard treatment for anorectal abscesses remains prompt incision and drainage, ideally preceded by an assessment of sphincter function, relevant surgical or obstetric history, anticoagulation status, and any associated urogenital conditions [45,56,57,58,59,60,61,62]. While most perianal and small ischiorectal abscesses can be managed safely under local anesthesia, larger or more complicated cases generally require operative drainage under appropriate anesthesia to ensure complete evacuation and reduce the risk of recurrence [63,64]. The choice of surgical approach depends on the abscess’s relation to the sphincter complex: incisions should be made as close to the anal verge as possible to minimize fistula formation, and careful probing, debridement, irrigation, and hemostasis are necessary to avoid premature wound closure and inadvertent nerve damage [65]. Under sedation and proper positioning, a digital rectal examination may reveal a fluctuant area suitable for drainage through the internal sphincter if needed, although some surgeons recommend extending the incision cephalad to or above the dentate line for better access [66].

Management of supralevator abscesses requires a precise anatomical understanding to select the safest drainage route: transrectal drainage is suitable for intersphincteric spread, transperineal drainage is indicated when infection extends through muscle planes, and percutaneous or transabdominal drainage may be appropriate if the abscess is associated with intra-abdominal pathology, guided by MRI when indicated [51,67,68].

Horseshoe abscesses, which originate in the deep postanal space and may extend unilaterally or bilaterally into the ischiorectal fossae, are best treated in the operating room using the modified Hanley technique. This approach involves division of the anococcygeal ligament, placement of strategic counter-incisions, and staged seton placement to ensure adequate drainage and reduce recurrence. In contrast, deep postanal abscesses without lateral spread can often be managed with an intersphincteric approach that allows direct drainage and simultaneous repair of the sphincter defect [11,69].

Evidence from a multicenter randomized trial of 98 patients showed that needle aspiration combined with clindamycin resulted in a significantly higher recurrence rate (41%) than standard incision and drainage (15%), confirming that needle aspiration should not replace conventional surgical drainage [70]. Small drainage catheters, such as de Pezzer or Malecot drains (12–14 Fr), have proven effective as adjuncts, offering patient comfort comparable to or better than traditional packing without increasing the risk of recurrence or fistula formation. Alternative techniques, including multiple counter-incisions with Penrose or vessel loop drains (commonly used in pediatric cases), have also demonstrated safety and efficacy in adults when initial evacuation is thorough [71,72,73,74].

Postoperative antibiotics are indicated selectively—for example, in cases with sepsis, extensive cellulitis, immunosuppression, or significant cardiac comorbidity—but their role in preventing fistula formation remains unclear, and current evidence does not support routine prophylaxis according to guidelines from the American Society of Colon and Rectal Surgeons [75,76,77,78].

While *Escherichia coli* is the most common pathogen in anorectal abscesses, followed by *Bacteroides* species, routine microbiological cultures are generally unnecessary as they rarely alter treatment plans. Cultures are recommended in special circumstances, such as suspected MRSA, immunosuppression, severe sepsis, or multidrug-resistant infections [79,80,81]. In neutropenic patients, who have an incidence of anorectal infection exceeding 10%, classic signs such as erythema or fluctuance may be absent due to impaired neutrophil response. In these patients, careful rectal examination and early empiric broad-spectrum antibiotics are essential, with drainage performed when a collection is confirmed either clinically or by imaging, or once hematologic recovery occurs [82,83].

The surgical management of a fused ischiorectal phlegmon with preperitoneal and retroperitoneal extension and associated purulent peritonitis requires urgent, aggressive intervention. This typically involves exploratory laparotomy with wide incision, complete evacuation of purulent collections, extensive peritoneal lavage, adequate drainage placement, and broad-spectrum antibiotic therapy, ideally adjusted according to culture results when available.

Anorectal abscesses accompanied by systemic signs such as fever must be closely monitored, as conservative treatment alone is often insufficient in advanced cases with preperitoneal spread. Early signs of retroperitoneal involvement—such as upper abdominal or lower back pain—should raise clinical suspicion. Anterior perforation may present with left flank tenderness or a palpable rectal mass. Notably, preperitoneal extension of perirectal abscesses, with intra-abdominal purulent collections, has been recognized since early surgical literature from 1942, with modern anatomical studies confirming the continuity between the preperitoneal, retrovesical, and perirectal spaces—particularly when early surgical intervention is delayed [45].

In cases of fused ischiorectal phlegmon with extensive extraperitoneal spread and sepsis, high clinical vigilance and early advanced imaging (preferably contrast-enhanced CT) are essential to delineate the full extent of disease. Prompt surgical intervention must be adapted to the anatomical complexity to minimize complications and reduce mortality.

The uniqueness of this condition lies in its rare and aggressive spread beyond the usual limits of the ischiorectal fossa, infiltrating deeper and less compartmentalized spaces. This atypical extension dramatically increases the risk of severe sepsis and multiorgan failure due to rapid, uncontrolled dissemination. Because the clinical presentation can be vague and nonspecific, delayed recognition is common, emphasizing the need for heightened awareness and timely imaging.

Successful management requires a comprehensive and multidisciplinary surgical approach that ensures complete drainage, meticulous debridement of necrotic tissue, and, when necessary, fecal diversion. Intensive postoperative monitoring and supportive critical care are vital to reduce the risk of rapid deterioration.

Due to the challenge of adequately draining deep extraperitoneal pockets, these cases carry a high risk of recurrence and severe septic complications. Early diagnosis and timely, aggressive surgical management remain crucial to improving outcomes in this rare but life-threatening condition.

Given the low incidence of preperitoneal and retroperitoneal extension in ischiorectal phlegmon, we emphasize the need for a clear clinical algorithm to guide a timely, structured, and multidisciplinary approach to anorectal sepsis. Such an algorithm should integrate early clinical assessment, targeted laboratory and imaging investigations, and prompt, appropriate surgical and medical interventions to prevent severe systemic complications (Table 4).

Current literature offers limited evidence regarding the pre- and retroperitoneal extension of ischiorectal phlegmon, underlining the lack of standardized management protocols and the urgent need for systematic research and evidence-based clinical guidelines (Table 5).

An earlier presentation to the emergency department, combined with prompt advanced imaging and timely surgical exploration, might have enabled an earlier diagnosis and intervention. Nevertheless, the deep and clinically silent progression of this phlegmon illustrates the substantial diagnostic challenge, highlighting the need for increased clinical awareness, thorough early assessment, and a coordinated multidisciplinary approach to optimize patient outcomes. This case report and literature review underline the diagnostic and surgical challenges of complex ischiorectal phlegmon and emphasize the need for prompt multidisciplinary management.

## 4. Conclusions

This case report and narrative review demonstrate how a seemingly silent ischiorectal phlegmon can progress to extensive preperitoneal and retroperitoneal involvement, leading to rapid septic deterioration even in the absence of local perianal signs. The fatal outcome, despite timely surgical intervention and broad-spectrum antibiotic therapy, underscores the diagnostic and therapeutic challenges of deep extraperitoneal spread. This highlights the need for early recognition, advanced imaging, and coordinated multidisciplinary management. Future research should focus on developing standardized, evidence-based guidelines for managing complex anorectal abscesses with deep compartment extension.

## Figures and Tables

**Table 1:** Timeline of the patient’s clinical course.

Day	Event
**−3**	Onset of diffuse abdominal pain, fever, chills
**0**	Emergency presentation, lab work, CT scan
**0**	Emergency midline laparotomy, drainage, debridement
**+0 h**	Post-op intensive care, broad-spectrum antibiotics
**+6 h**	Cardiorespiratory arrest—unsuccessful resuscitation

Legend: CT = computed tomography.

**Table 2:** Laboratory tests upon admission.

Laboratory Tests	Laboratory Tests Upon Admission	Reference Range
***White blood cell count***	19.20 × 10^3^ cells/μL	4.0–10.0 ×10^3^/µL
***Neutrophil proportion***	89.8%	40–75%
***Hemoglobin***	14.1 g/dL	12–16 g/dL
***Platelet count***	124 × 10^3^ cells/μL	150–400 ×10^3^/µL
***Creatinine***	1.7 mg/dL	0.6–1.2 mg/dL
***Urea***	93 mg/dL	15–50 mg/dL
***INR***	1.21	0.8–1.2
***Prothrombin time***	14.3 sec	11–13.5 sec
***Fibrinogen***	1115 mg/dL	200–400 mg/dL
***C-reactive proteine***	30 mg/dL	<5 mg/dL
***Erythrocyte sedimentation rate***	55 mm/h	0–20 mm/h
***Procalcitonin***	3 ng/mL	<0.05–0.1 ng/mL
***Serum Lactate***	4 mmol/L	0.5–1.5 mmol/L

**Table 3:** A narrative review of spreading anorectal abscesses, illustrated by the addition of a newly reported case.

Study	Type of Abscess	Abdominal Pain	Comorbidities	Clinical Presentation	Site of Expansion	Treatment
**Darlington and Anitha [57]**	Ischiorectal	Yes	Uncontrolled Diabet mellitus	Abdominal wall cellulitis and swealing and pain	Preperitoneal	Stab incision
**Okuda [58]**	Perianal	Yes	Not mentioned	Perianal swelling and pain	Retroperitoneal	Simple drainage + LMEI with primary closure
**Butt [59]**	Ischiorectal horseshoe	Yes	Uncontrolled Diabet mellitus	Perianal swelling and pain	Pre-and retroperitoneal	Simple drainage
**Hamza [60]**	Perianal horseshoe	Yes	Not mentioned	Perianal swelling and pain	Preperitoneal	Simple drainage + LMEI with primary closure
**Mentzer [61]**	Perirectal	Yes	Not mentioned	Perianal swelling and pain	Pre-and retroperitoneal	LMEI with VAC
**Pehlivanli [44]**	Ischiorectal horseshoe	Yes	Not mentioned	Perianal swelling and pain	Retroperitoneal	Simple drainage
**Alzaz [62]**	Ichiorectal	Yes	Uncontrolled Diabet mellitus	Perianal swelling and pain	Retroperitoneal	Laparotomy, Perianal drainage
**Papadopoulos [45]**	Perirectal	Yes	Diabet mellitus, Gout and myocardial infarction	Perianal swelling and pain	Pre-and retroperitoneal	A right gluteal incision and intrasphincteric drainage
**Oikonomou [46]**	Supralevator horse shoe	Yes	Not mentioned	Perianal swelling and pain	Preperitoneal	Drainage, abdominal incisionfistulotomy, colostomy
**Our case**	Ischiorectal horseshoe	Yes	Not mentioned	Generalized muscular guarding	Pre-and retroperitoneal	Laparotomy, Incison an peritoneal drainage

Legend: LMEI = Lower Midline Extraperitoneal Incision, VAC = Vacuum-Assisted Closure.

**Table 4:** Structured Approach to Anorectal Sepsis: Diagnostic and Therapeutic Framework.

Clinical Assessment	Check History, Physical Exam, and Vital Signs.
Initial Stabilization	Begin IV fluids, oxygen, and broad-spectrum antibiotics.
Lab Tests	Order CBC, CRP, procalcitonin, lactate, and obtain cultures.
Imaging	Use perianal ultrasound, pelvic CT, or MRI as needed.
Team Decision	Involve surgery, ICU, and infectious disease; drain abscesses early.
Monitoring	Track vitals and labs, adjust treatment, and repeat imaging if necessary.

Legend: IV = Intravenous, CBC = Complete Blood Count, CRP = C-Reactive Protein, CT = Computed Tomography, MRI = Magnetic Resonance Imaging, ICU = Intensive Care Unit.

**Table 5:** Identified Gaps in Research on Ischiorectal Phlegmon with Pre- and Retroperitoneal Spread.

Rare occurrence and limited documentation	The extremely low incidence and sparse documentation of such cases make it challenging to establish clear evidence-based recommendations and highlight the need for further systematic reporting
Absence of prospective or controlled studies	Variations in pelvic anatomy, patient comorbidities, and diverse clinical presentations continue to complicate the development of uniform, evidence-based treatment strategies.
Anatomical and clinical variability	Anatomical variations, patient comorbidities, and heterogeneous clinical presentations continue to hinder the establishment of clear, standardized management guidelines.
Delayed or difficult diagnosis	Non-specific symptoms and restricted access to advanced imaging in certain healthcare settings often lead to underdiagnosis and delayed intervention.
Lack of validated treatment protocols	There are no universally accepted guidelines, and current management frequently relies on institutional protocols or the clinician’s individual judgment.

## Data Availability

The data presented in this study are available on request from the corresponding author. The data are not publicly available due to patient confidentiality.
